# Targeted Elimination of G Proteins and Arrestins Defines Their Specific Contributions to Both Intensity and Duration of G Protein-coupled Receptor Signaling*

**DOI:** 10.1074/jbc.M116.754887

**Published:** 2016-11-16

**Authors:** Elisa Alvarez-Curto, Asuka Inoue, Laura Jenkins, Sheikh Zahir Raihan, Rudi Prihandoko, Andrew B. Tobin, Graeme Milligan

**Affiliations:** From the ‡ Centre for Translational Pharmacology, Institute of Molecular, Cell and Systems Biology, College of Medical, Veterinary and Life Sciences, University of Glasgow, Glasgow G12 8QQ, Scotland, United Kingdom,; the § Graduate School of Pharmaceutical Sciences, Tohoku University, Sendai, Miyagi 980-8578, Japan, and; the ¶ Japan Science and Technology Agency (JST), Precursory Research for Embryonic Science and Technology (PRESTO), Kawaguchi, Saitama 332-0012, Japan

**Keywords:** arrestin, calcium intracellular release, CRISPR/Cas, extracellular-signal-regulated kinase (ERK), fatty acid, G protein, G protein-coupled receptor (GPCR)

## Abstract

G protein-coupled receptors (GPCRs) can initiate intracellular signaling cascades by coupling to an array of heterotrimeric G proteins and arrestin adaptor proteins. Understanding the contribution of each of these coupling options to GPCR signaling has been hampered by a paucity of tools to selectively perturb receptor function. Here we employ CRISPR/Cas9 genome editing to eliminate selected G proteins (Gα_q_ and Gα_11_) or arrestin2 and arrestin3 from HEK293 cells together with the elimination of receptor phosphorylation sites to define the relative contribution of G proteins, arrestins, and receptor phosphorylation to the signaling outcomes of the free fatty acid receptor 4 (FFA4). A lack of FFA4-mediated elevation of intracellular Ca^2+^ in Gα_q_/Gα_11_-null cells and agonist-mediated receptor internalization in arrestin2/3-null cells confirmed previously reported canonical signaling features of this receptor, thereby validating the genome-edited HEK293 cells. FFA4-mediated ERK1/2 activation was totally dependent on G_q_/_11_ but intriguingly was substantially enhanced for FFA4 receptors lacking sites of regulated phosphorylation. This was not due to a simple lack of desensitization of G_q_/_11_ signaling because the G_q_/_11_-dependent calcium response was desensitized by both receptor phosphorylation and arrestin-dependent mechanisms, whereas a substantially enhanced ERK1/2 response was only observed for receptors lacking phosphorylation sites and not in arrestin2/3-null cells. In conclusion, we validate CRISPR/Cas9 engineered HEK293 cells lacking G_q_/_11_ or arrestin2/3 as systems for GPCR signaling research and employ these cells to reveal a previously unappreciated interplay of signaling pathways where receptor phosphorylation can impact on ERK1/2 signaling through a mechanism that is likely independent of arrestins.

## Introduction

In recent years the concept that G protein-coupled receptors (GPCRs)^3^ mediate their effects exclusively via activation of one or more members of the family of heterotrimeric G proteins has been shown to be incorrect (1). Although such canonical signaling via G proteins is integral to the regulation of second messenger production, a substantial number of intracellular adaptor proteins can also interact either directly or as part of larger protein complexes with GPCRs to modify downstream signal transduction and control physiological functions. The most studied of such adaptor proteins are members of the arrestin family (2–4), and it is clear despite being named originally for their capacity to “arrest” and, therefore, block G protein-mediated signaling that they can regulate positively many cellular functions in a GPCR-dependent manner (2–4). A great deal of our understanding of the roles of arrestin2 and arrestin3 (frequently still designated β-arrestin1 and β-arrestin2, respectively) has derived either from studies employing arrestin isoform-null mice (5, 6) and tissues and cells derived from them or, in the absence of useful chemical inhibitors of the arrestins, the application of siRNA and related technologies to reduce steady-state levels of these proteins. Although useful, the inability to completely abolish expression of arrestins in such “knockdown” studies compromises quantitative interpretation. This is particularly so given that the end points of arrestin signaling are often downstream of highly amplified signal transduction pathways, for example within intracellular serine/threonine kinase cascades. Thus, knockdown experiments that leave even a modest amount of arrestin expression may still maintain sufficient amounts of an arrestin to produce a near full signal or function. An alternative strategy is to use mouse embryo fibroblasts derived from arrestin-null animals (7–9), but these cells are challenging to transfect, limiting their usefulness. This has meant that despite the caveats described above and the fact that such arrestin-null mouse embryo fibroblasts have shown that, in this cell type, activation of the extracellular signal-regulated kinases ERK1/2 by certain ligands at the β_2_-adrenoreceptor requires an arrestin (10), much of the basis of underpinning analysis of arrestin signaling has derived from studies in more tractable, transformed cell lines, including human embryonic kidney (HEK) 293 cells where arrestin levels have been reduced but not completely eliminated (11–14).

In an effort to define more fully specific roles of G protein *versus* arrestin signaling in response to activation of free fatty acid receptor 4 (FFA4, also called GPR120) (15, 16), we employed CRISPR/Cas9-mediated genome-editing (17, 18) to produce HEK293 cell clones that are null for either Gα_q_ and Gα_11_, the pair of G proteins that transmit receptor activation to phosphoinositidase C and thence the elevation of intracellular Ca^2+^ (19, 20), or are null for both arrestin2 and arrestin3. Each of these lines was then further transfected to stably express either wild type FFA4 or a form of this receptor that cannot be phosphorylated in response to an agonist ligand because each of the residues in the C-terminal tail that becomes phosphorylated in the wild type receptor has been mutated to alanine (21, 22). We show that either restricting interaction of FFA4 with arrestins via this mutational strategy or eliminating expression of the arrestins results in prolongation of Ca^2+^ signaling via FFA4, whereas we also show that arrestins do not contribute directly to FFA4-mediated ERK1/2 MAP kinase phosphorylation/activation in HEK293 cells. Rather, with a phosphorylation-deficient form of FFA4, agonist regulation of ERK1/2 phosphorylation is markedly enhanced in the absence or presence of arrestins. By contrast, in cells lacking expression of G_q_/G_11_ or by chemical inhibition of these G proteins, the FFA4 receptor fails to activate this pathway (23).

## Results

### 

#### 

##### Characterization of HEK293 Cells Lacking Gα_q_ and Gα_11_ or Arrestin2 and Arrestin3

**FIGURE 1.**
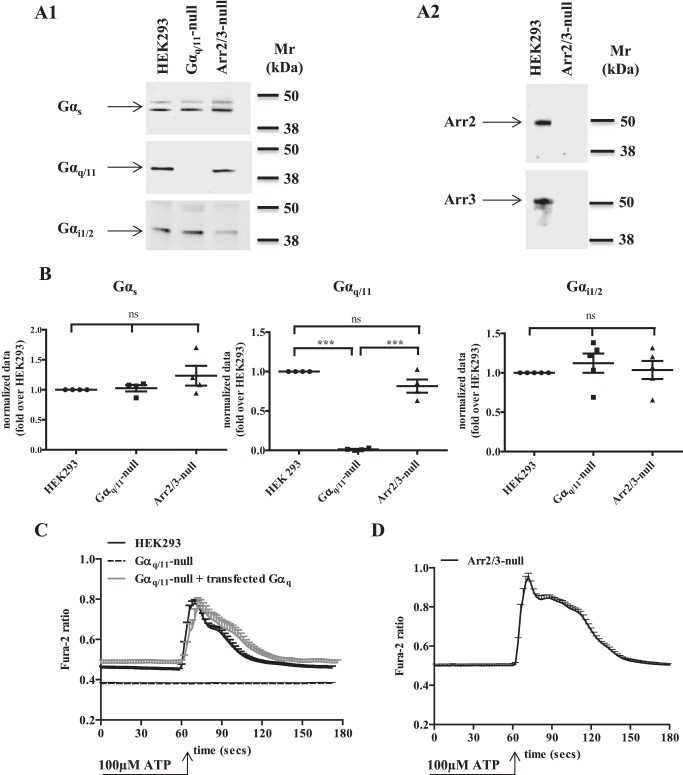
**Characteristics of Gα_q_/Gα_11_ and arrestin2/3-null HEK293 cells.** Immunoblotting studies were performed on (*A1*) membrane preparations of parental HEK293 cells and clones from genome-edited cells that eliminate expression of Gα_q_ + Gα_11_ or arrestin2 + arrestin3. Such samples were probed to detect expression of the long and short isoforms of Gα_s_, Gα_q_ + Gα_11_, and Gα_i1/2_. *A2*, cytosolic preparations from wild type HEK293 cells and arrestin2/3-null cells were immunoblotted to detect expression of arrestin2 (*upper panel*) or arrestin3 (*lower panel*). Representative immunoblots are displayed in both *A1* and *A2. B*, a series of such immunoblots was quantified by densitometric analysis. Individual results of immunoblots taken from at least four separate cell preparations are shown relative to levels detected in parental HEK293 cells. Values from individual experiments are shown as the group means ± S.E. *ns* = not significantly different; ***, different at *p* < 0.001. *C*, single cell Ca^2+^ imaging was performed on parental HEK293 cells and on both Gα_q_/Gα_11_-null cells and such cells into which Gα_q_ had been re-introduced. ATP (100 μm) was added at the indicated time. *D*, studies akin to those in *C* were performed in arrestin2/3-null cells. ATP (100 μm) was added at the indicated time.

CRISPR/Cas9-mediated genome-editing was used to eliminate expression from HEK293 cells of either the α subunits of both of the phosphoinositidase C-activating G proteins G_q_ and G_11_ or of both the ubiquitously expressed arrestin isoforms, arrestin2 and arrestin3. Immunoblotting studies performed on membranes from cells selected to lack expression of both Gα_q_ and Gα_11_ showed that although neither of these polypeptides could be detected (Fig. 1, *A1* and *1B*), this procedure and selection process did not affect significantly expression of the α subunits of other G protein subtypes, including the long and short isoforms of Gα_s_ and Gα_i_ family members (Fig. 1, *A1* and *1B*). Similarly, immunoblotting of cytosolic preparations from cells selected to lack expression of both arrestin2 and arrestin3 confirmed that neither of these proteins could now be detected, although both were present in the parental HEK293 cells (Fig. 1, *A2*). Moreover, elimination of the arrestins also did not significantly affect expression of G proteins, including Gα_q_/Gα_11_ (Fig. 1, *A1* and *1B*). As shown previously (23), ATP, acting at a P2Y purinoceptor expressed endogenously by HEK293 cells, was unable to cause elevation of [Ca^2+^]*_i_* in Gα_q_/Gα_11_-null cells (Fig. 1*C*), although this was easily measured in single cell Ca^2+^ imaging studies performed on parental HEK293 cells (Fig. 1*C*) and was robustly recapitulated by reintroduction of Gα_q_ into the Gα_q_/Gα_11_-null cells (Fig. 1*C*). By contrast, ATP was able to generate a robust Ca^2+^ response in arrestin2/3-null cells (Fig. 1*D*).

**FIGURE 2.**
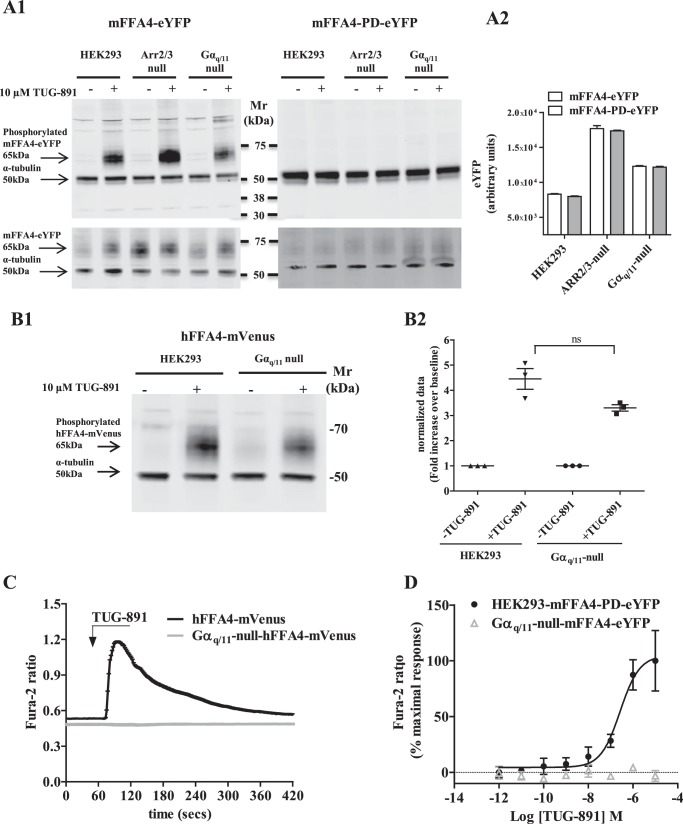
**Wild type but not PD forms of FFA4 became phosphorylated on amino acids Thr^347^ and Ser^350^ upon the addition of agonist.** Parental, Gα_q_/Gα_11_-null and arrestin2/3-null HEK293 cells stably expressing either mFFA4-eYFP (*left-hand side*) or mFFA4-PD-eYFP (*right-hand side*) were stimulated with vehicle or TUG-891 (10 μm, 5 min). Subsequently, cell lysates were resolved by SDS-PAGE and immunoblotted with a mixture of (mouse FFA4 specific) anti-phospho Thr^347^-Ser^350^ (22) and anti-α-tubulin antibodies, detecting the specific 65-kDa and 50-kDa polypeptides corresponding to the receptor and α-tubulin bands respectively (*A1*, *upper panels*). Parallel immunoblots were probed with a mixture of anti-GFP (recognizes also eYFP) and anti-α-tubulin antibodies (*A1*, *lower panels*). The higher level of TUG-891 mediated mFFA4-eYFP phosphorylation in the arrestin2/3-null HEK293 cells reflects the higher level expression of mFFA4 receptor protein in this clone (*A1*, *lower panel*). This was confirmed by assessment of eYFP fluorescence in cells in each clone (*A2*) and was also the case for mFFA4-PD-eYFP expressed in the arrestin2/3-null background (*A2*). *B*, parental and Gα_q_/Gα_11_-null HEK293 cells expressing hFFA4-mVenus were also stimulated with vehicle or TUG-891 (10 μm, 5 min). Phosphorylation of the receptor was detected with a human FFA4 specific anti-phospho Thr^347^-Ser^350^ antiserum (21), and the co-addition of anti-α-tubulin antibodies provided loading controls (*B1*). *B2*, quantification of the extent of agonist-induced phosphorylation was produced by densitometric analysis of three experiments, each performed on samples derived from separate cell treatments: *ns* = not significantly different. *C*, HEK293 and Gα_q_/Gα_11_-null cells expressing hFFA4- mVenus were challenged with 3 μm TUG-891 in single-cell imaging Ca^2+^ experiments. *D*, calcium mobilization experiments were performed on populations of parental HEK293 and Gα_q_/Gα_11_-null cells expressing the mFFA4-eYFP construct. These demonstrated that mFFA4-mediated Ca^2+^ release was lost in the absence of Gα_q_/Gα_11_.

We recently defined the sites of agonist-regulated phosphorylation within the C-terminal tail of both mouse (m)FFA4 and human (h)FFA4 and defined that conversion of these serine and threonine residues to alanines produces phosphorylation-deficient (PD) forms of the receptor orthologs (21, 22). We also recently proposed that detection of agonist-regulated GPCR phosphorylation using phospho-specific antibodies could be used as a biomarker for receptor activation (24). Here we used phospho-specific antibodies against the agonist-regulated phosphorylation sites Thr^347^ and Ser^350^ (21, 22) as a marker for FFA4 activation in genome-edited HEK293 cells. After stable expression of mFFA4-eYFP in each of parental HEK293 cells and the Gα_q_/Gα_11_ or arrestin2/3 genome-edited cell lines and selection of individual clones, activation of mFFA4 by the agonist TUG-891 (25–27) was produced no-matter the genetic status of the cells (parental or genome-edited) (Fig. 2*A1*, *upper panels*). Internal loading controls for such studies were provided by concurrent detection of levels of α-tubulin that migrates in SDS-PAGE at a position akin to a 50-kDa marker protein (Fig. 2*A1*, *upper panels*). The selectivity of the FFA4 phospho-specific antibodies used to assess the activation status of FFA4 was confirmed by the lack of detection of mFFA4-PD-eYFP, a variant of the receptor lacking phosphorylation sites including Thr^347^ and Ser^350^ (Fig. 2*A1*, *upper panels*). Measures of the total expression of mFFA4-eYFP and mFFA4-PD-eYFP, assessed either by immunoblotting with an anti-eYFP antiserum (Fig. 2*A1*, *lower panels*) or by measuring fluorescence corresponding to enhanced yellow fluorescent protein (*eYFP*; Fig. 2*A2*), showed that each receptor variant was present at similar levels in parental and Gα_q_/Gα_11_-null HEK293 cells but at a somewhat higher level in the arrestin2/3-null cells. hFFA4 tagged at the C terminus with mVenus (a variant of eYFP) also showed similar activation by TUG-891 when expressed stably in either parental or Gα_q_/Gα_11_-null HEK293 cells (Fig. 2*B*). Thus, both mouse and human FFA4 were activated to promote receptor phosphorylation in a similar manner by agonist in parental, Gα_q_/Gα_11_-null and arrestin2/3-null cells.

### Distinguishing the Dependence of FFA4 Signaling Mediated via G_q/11_, Arrestin2/3, and Receptor Phosphorylation

#### 

##### Coupling to Intracellular Calcium Mobilization

Single cell Ca^2+^ experiments showed a sharp peak of calcium release upon agonist stimulation of hFFA4-mVenus in parental cells but not in Gα_q_/Gα_11_-null cells (Fig. 2*C*). These data are consistent with our previous studies that established FFA4 coupling to increases in intracellular calcium through canonical G_q/11_-dependent activation of phosphoinositidase C. Further validation of the loss of canonical G_q/11_ signal transduction in the G_q/11_-null cells was established by the lack of calcium response to stimulation of mFFA4-PD-eYFP (a receptor variant uncoupled from receptor phosphorylation/arrestin signaling) when expressed stably in Gα_q_/Gα_11_-null cells (Fig. 2*D*) and defined that Ca^2+^ signaling is independent of receptor phosphorylation/arrestin signaling.

##### Coupling to Inositol Phosphate Signaling

**FIGURE 3.**
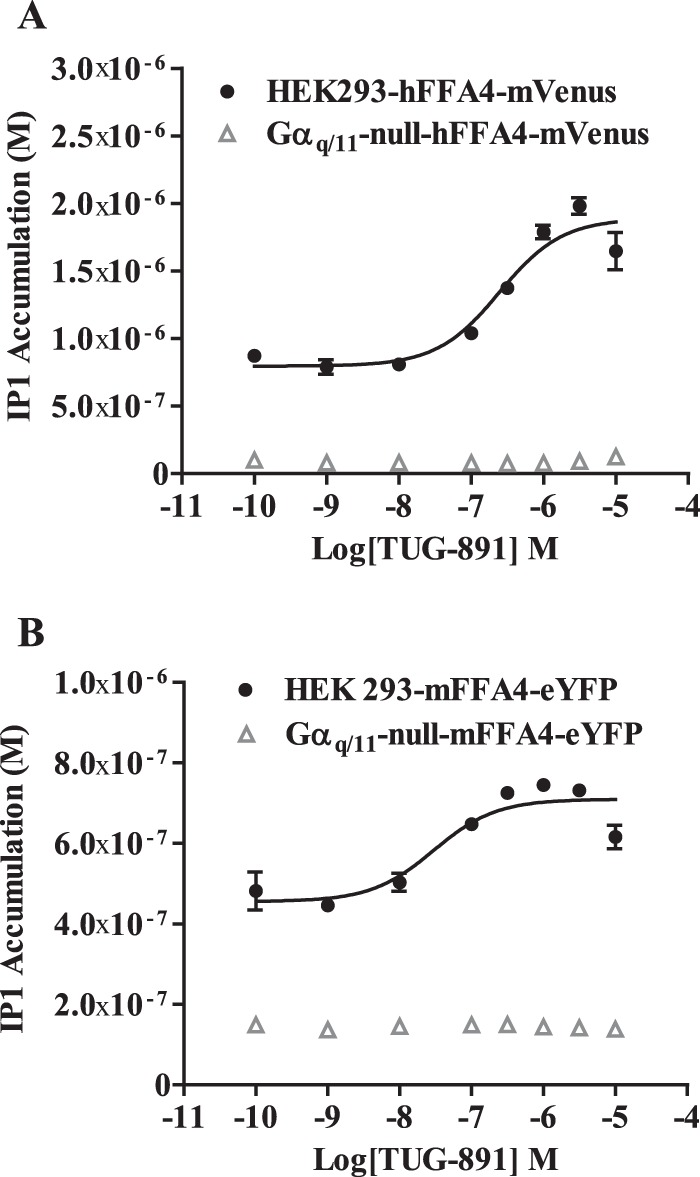
**FFA4-mediated inositol phosphate generation is lacking in Gα_q_/Gα_11_-null HEK293 cells.** Parental and Gα_q_/Gα_11_-null HEK293 cells stably expressing human (*A*) or mouse (*B*) FFA4 were used to measure basal and TUG-891-mediated levels of inositol monophosphates.

As anticipated from recognition that Gα_q_ and Gα_11_ are key transducers of phosphoinositidase C signaling in HEK293 cells, upon the addition of TUG-891 wild type forms of both hFFA4 and mFFA4 promoted enhanced accumulation of inositol monophosphates (IP1) in parental HEK293 cells expressing either hFFA4 (Fig. 3*A*) or mFFA4 (Fig. 3*B*). However, this did not occur for the receptor orthologs expressed in Gα_q_/Gα_11_-null HEK293 cells (Fig. 3, *A* and *B*), consistent with the lack of elevation of intracellular Ca^2+^ in this situation. Notably, the basal level of IP1 was lower in Gα_q_/Gα_11_-null HEK293 cells expressing either human or mouse FFA4 than in equivalent parental HEK293 cells (Fig. 3, *A* and *B*), indicative of a degree of constitutive activation of the G_q_/G_11_-phosphoinositidase C signaling axis for both receptor orthologs.

##### Receptor Internalization

**FIGURE 4.**
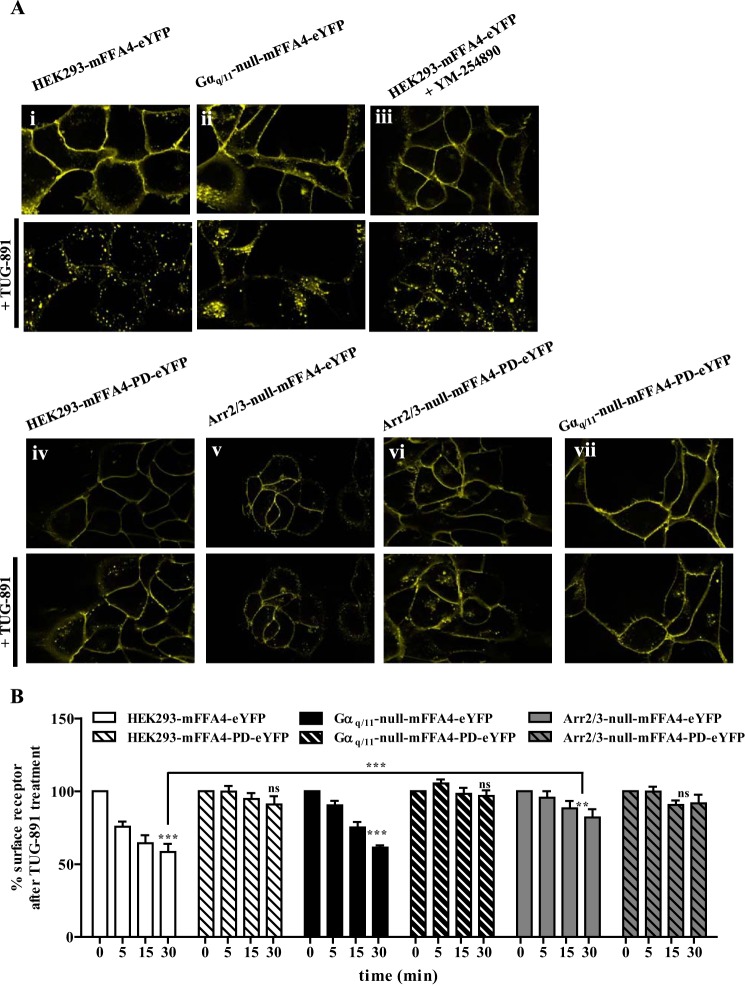
**Effects of elimination of Gα_q_ + Gα_11_ or arrestin2 + arrestin3 on agonist-induced internalization of FFA4.** *A*, parental (*i*, *iii*, and *iv*), Gα_q_/Gα_11_-null (*ii* and *vii*), or arrestin2/3-null (*v* and *vi*) HEK293 cells stably expressing mFFA4-eYFP (*i–iii* and *v*) or mFFA4-PD-eYFP (*iv* and *vi–vii*) were exposed to TUG-891 (10 μm) (*upper panels*, *t* = 0; *lower panels*, *t* = 30 min). In *iii*, the Gα_q_/Gα_11_ inhibitor YM-254890 (100 nm) was added 30 min before the addition of TUG-891. Representative images of experiments performed at least three times are shown. *B*, cell surface ELISA experiments were performed to detect the FLAG-epitope tag introduced into the extracellular N-terminal domain on each form of FFA4 at various times after the addition of TUG-891. 100% represents receptor at the cell surface before the addition of TUG-891. Agonist-induced internalization was observed at the 30-min time point (**, *p* < 0.01; ***, *p* < 0.001). The extent of internalization of mFFA4-eYFP was greater (*p* < 0.001) in parental than in arrestin2/3-null HEK293 cells. *ns* = not significantly different from *t* = 0.

**FIGURE 5.**
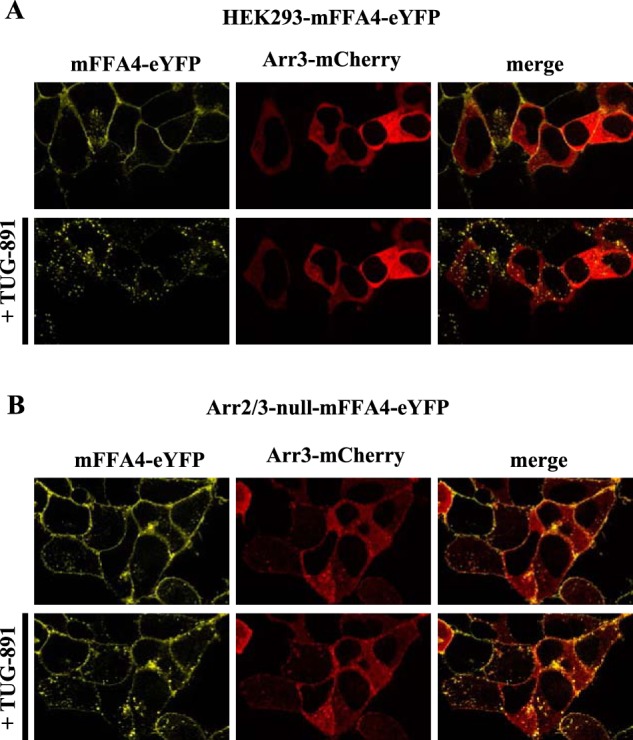
**Reintroduction of arrestin3 into arrestin2/3-null HEK293 cells restored agonist-mediated internalization of FFA4.** Parental (*A*) or arrestin2/3-null (*B*) HEK293 cells stably expressing mFFA4-eYFP were transfected transiently to express arrestin3-mCherry and treated with TUG-891 (10 μm) (*upper panels*: *t* = 0; *lower panels*: *t* = 30 min). Representative images of the location of mFFA4-eYFP (*yellow channel*) and arrestin3-mCherry (*red channel*) are shown. In the *right-hand panels* these images are merged to provide color overlap.

In each cell line expressing either wild type mFFA4 or mFFA4-PD the introduced receptor was directed effectively to the surface of the cells (Fig. 4). As anticipated from previous studies, the addition of the FFA4 agonist TUG-891 resulted in internalization of mFFA4-eYFP in parental HEK293 cells that was both rapid and extensive (Fig. 4, *Ai*). This was also the case for mFFA4-eYFP expressed in cells lacking Gα_q_/Gα_11_ (Fig. 4, *Aii*). As anticipated from this observation, pretreatment of parental HEK293 cells expressing mFFA4-eYFP with the Gα_q_/Gα_11_ inhibitor YM-254890 (28–31) also did not affect agonist-induced internalization of the receptor (Fig. 4, *Aiii*). In contrast, when mFFA4-PD-eYFP was expressed in parental HEK293 cells agonist-induced internalization of the receptor was greatly reduced (Fig. 4, *Aiv*). Moreover, when either wild type (Fig. 4, *Av*) or mFFA4-PD-eYFP (Fig. 4, *Avi*) was expressed in arrestin2/3-null HEK293 cells, agonist-induced internalization of the receptor appeared to be all but lacking. This was also the case for mFFA4-PD-eYFP expressed in Gα_q_/Gα_11_-null cells (Fig. 4, *Avii*). Cell surface enzyme-linked immunosorbent assay (ELISA) studies detecting the FLAG epitope tag incorporated into the N-terminal region of each receptor construct provided quantitation of these effects over time (Fig. 4*B*). In support of the cell imaging studies, extensive and time-dependent reductions in cell surface mFFA4-eYFP were observed in both parental and Gα_q_/Gα_11_-null HEK293 cells. In contrast, internalization of mFFA4-eYFP expressed in arrestin2/3-null HEK293 cells (Fig. 4*B*) was not evident at early time points of agonist stimulation with only a small response observed after 30 min of stimulation (Fig. 4*B*). These data support the notion that FFA4 internalization is largely dependent on arrestin2/3. Interestingly, removal of the phosphorylation sites from FFA4, a process that almost completely uncouples the receptor from arrestin interactions (21, 22), totally prevented receptor internalization (Fig. 4*B*). These data suggest that FFA4 internalization is independent of G_q_/_11_ signaling but, rather, is completely dependent on receptor phosphorylation, which acts primarily to drive receptor internalization through an arrestin-dependent pathway, although there is a minor component that is arrestin-independent. To further validate the arrestin2/3-null cells as a tool to dissect the role of arrestins in the internalization of FFA4, we restored TUG-891-induced FFA4 internalization in arrestin2/3-null cells by transiently expressing an arrestin3-mCherry fluorescent protein construct in these cells (Fig. 5).

##### G_q_/_11_-mediated Calcium Responses Are Desensitized through Both Receptor Phosphorylation and Arrestin-dependent Mechanisms

**FIGURE 6.**
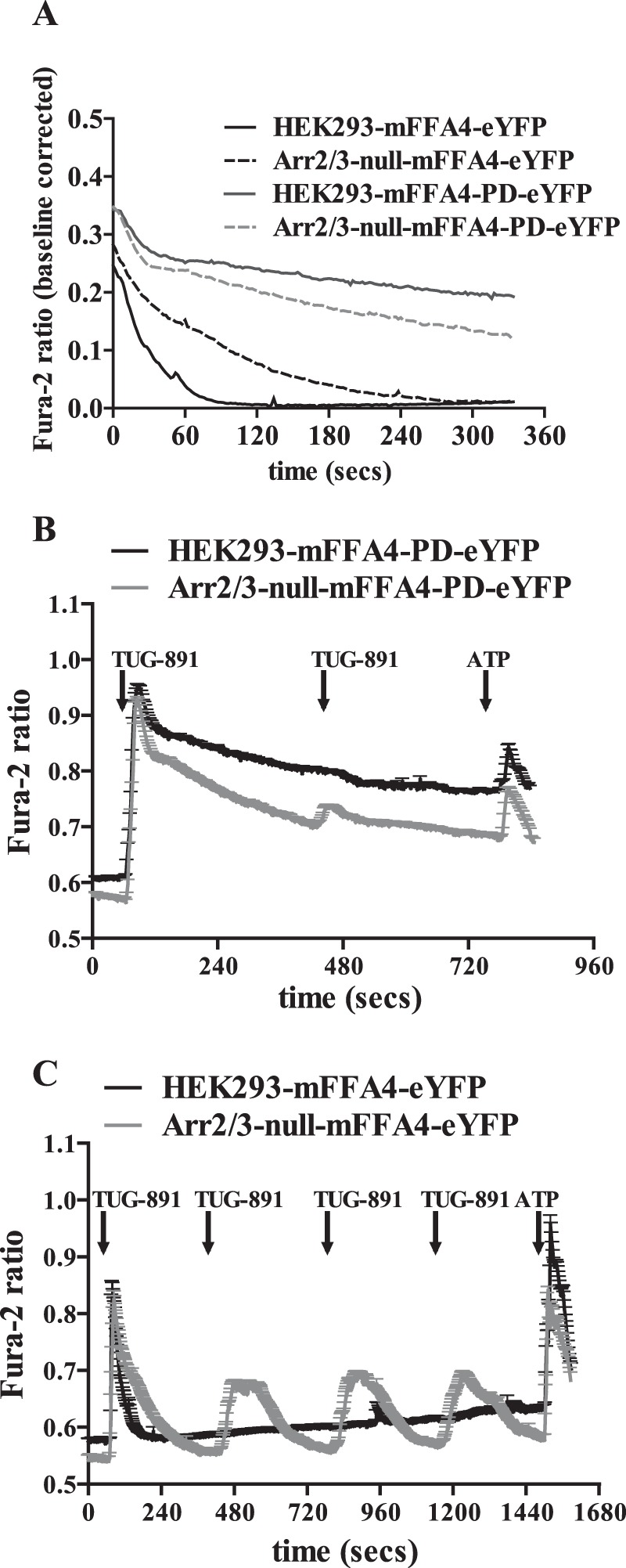
**Lack of arrestin2/3 and resistance to agonist-induced receptor phosphorylation extended the kinetics of Ca^2+^ elevation in HEK293 cells.** Intracellular Ca^2+^ levels and how these were affected over time by TUG-891-induced activation of FFA4 were assessed in a series of single cell Ca^2+^ imaging studies performed in both parental and arrestin2/3-null HEK293 cells expressing either wild type or mFFA4-PD as indicated. *A*, representative traces in parental HEK293 cells expressing wild type mFFA4 (*HEK293-mFFA4-eYFP*), parental cells expressing mFFA4-PD (*HEK293-mFFA4-PD-eYFP*), arrestin2/3-null cells expressing wild type mFFA4 (*Arr2/3-null-mFFA4-eYFP*), and arrestin2/3-null cells expressing mFFA4-PD (*Arr2/3-null-mFFA4-PD-eYFP*). *B*, shows data from studies on parental and arrestin2/3-null HEK293 cells expressing mFFA4-PD in which after the addition and washout of two treatments with TUG-891 ATP was added at 760 s to activate endogenously expressed P2Y receptors. *C*, a single exposure to TUG-891 was sufficient to fully desensitize mFFA4 in parental HEK293 cells but not in arrestin2/3-null cells. Time(s) of addition of TUG-891 is shown as are subsequent additions of ATP.

**FIGURE 7.**
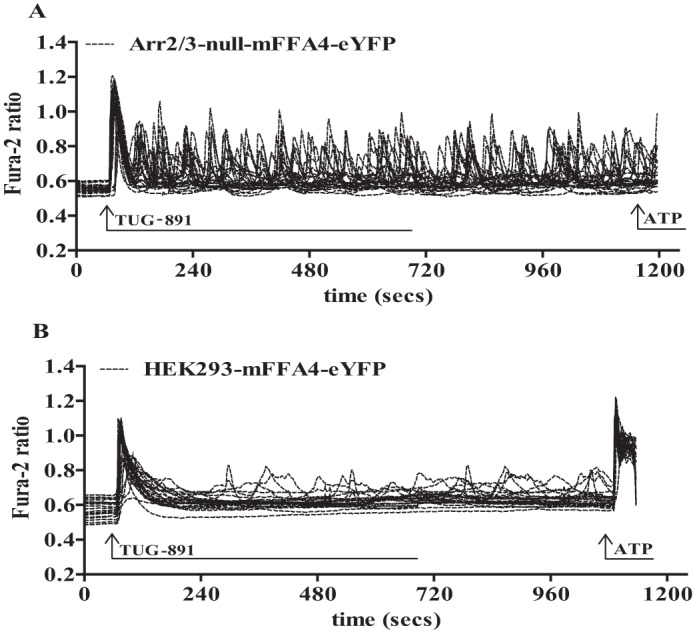
**Repetitive Ca^2+^ spiking in arrestin2/3-null HEK293 cells reflected a lack of FFA4 desensitization.** Arrestin2/3-null (*A*) and parental (*B*) HEK293 cells expressing mFFA4-eYFP were exposed to a single, maintained concentration of TUG-891 (3 μm) at the indicated point. Studies across 25 cells analyzed in single-cell Ca^2+^ imaging studies were overlaid. At the indicated time ATP (100 μm) was added.

We next considered regulation of [Ca^2+^]*_i_* and the contribution of arrestins and/or receptor phosphorylation to the kinetics and potential desensitization of FFA4. As highlighted, short term treatment of parental HEK293 cells expressing mFFA4-eYFP with TUG-891 resulted in rapid elevation of [Ca^2+^]*_i_*, an effect that declined in an essentially mono-exponential fashion, as Ca^2+^ was re-sequestered into intracellular stores (Fig. 6*A*). This occurred with a halftime of some 21.5 s (Table 1). In cells lacking arrestins but expressing wild type mFFA4-eYFP, after an equally rapid elevation of [Ca^2+^]*_i_* upon the addition of TUG-891, the kinetics of [Ca^2+^]*_i_* decline was substantially slower (halftime 66.6 s) (Fig. 6*A*, Table 1). Very extended kinetics of elevated [Ca^2+^]*_i_* and very slow decline toward basal levels were recorded after the addition of TUG-891 to both wild type HEK293 cells expressing mFFA4-PD-eYFP and in arrestin2/3-null cells expressing mFFA4-PD-eYFP (Fig. 6*A*, Table 1). Indeed, in these two final situations where the PD form of the mFFA4 receptor was expressed, the addition of ATP 12 min after the addition of TUG-891 was able to induce only a very modest further elevation of [Ca^2+^]*_i_* because levels still remained markedly elevated over basal at this time (Fig. 6*B*). This was in contrast to parental HEK293 cells expressing wild type mFFA4-eYFP or, indeed, arrestin2/3-null cells expressing wild type mFFA4-eYFP where, because Ca^2+^ levels had returned to basal, the addition of ATP now produced as robust a response as did the initial application of TUG-891 (Fig. 6*C*). Equally of note: although a single application of TUG-891 in parental HEK293 cells was able to largely desensitize mFFA4-eYFP to subsequent additions of the agonist after washout, in arrestin2/3-null cells response to TUG-891 did not desensitize across multiple washes and re-additions of the ligand (Fig. 6*C*). Analysis of these signals in the arrestin2/3-null cells showed that the initial peak of Ca^2+^ did, however, reflect a greater extent of release than in the parental HEK293 cells, which possibly reflects the extended kinetics of Ca^2+^ elevation in this cellular background (Table 2). Further peaks displayed similar, but a somewhat smaller, extent of release than the initial peak (Table 2). After a single addition of TUG-891 in arrestin2/3-null cells expressing wild type FFA4-eYFP, multiple individual spikes of Ca^2+^ release were produced in a repeated and non-synchronized manner across individual cells analyzed (Fig. 7*A*). This was not observed in parental HEK293 cells expressing wild type mFFA4-eYFP. where such repetitive spiking of Ca^2+^ release was largely absent (Fig. 7*B*).

**TABLE 1:** **Kinetics of Ca^2+^ elevation in genome-edited HEK293 cells** The speed at which the initial peak of Ca^2+^ decays over time was analyzed and compared between the four cell lines using a one-phase decay equation (Fig. 6*A*). From this half-life (*t*½) was assessed as a measure of how rapidly the signal declined over time and the Tau constant, as a measure of the rate constant for this change.

Cell line	*t*_1/2_	Tau constant
	*s*	*s*
HEK293-mFFA4-eYFP	21.5	30.9
HEK293-mFFA4-PD-eYFP	497.2	717.3
Arr2/3-null-mFFA4-eYFP	66.6	96.1
Arr2/3-null-mFFA4-PD-eYFP	259.2	374.0

**TABLE 2:** **Comparison of the area under the curve from single-cell Ca^2+^ experiments** Traces from cells illustrated in Fig. 6*C* were analyzed and subsequently compared by calculating the area under the curve of the first and only peak in the case of the HEK293-mFFA4-eYFP and the initial and subsequent peaks in the Arr2/3-null-mFFA4-eYFP expressing cells.

Cell line	HEK293 Initial peak	Arr2/3-null Initial peak	Arr2/3-null peak 2	Arr2/3-null peak 3	Arr2/3-null peak 4
Total area (ratio × s)	10.1	26.0	20.5	20.8	19.3

##### Complexity of FFA4 Activation of ERK1/2 Signaling Revealed by Genome-edited HEK293 Cells

**FIGURE 8.**
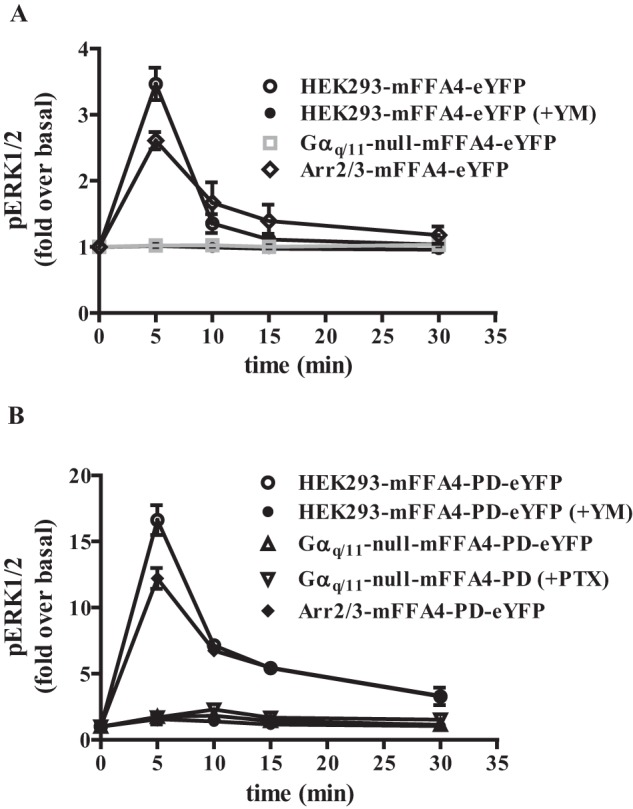
**FFA4-PD generated enhanced activation of ERK1/2 MAP kinase phosphorylation largely via Gα_q_/Gα_11_-mediated signals.** ERK1/2 phosphorylation studies were performed using wild type (*A*) or mFFA4-PD-eYFP (*B*) stably expressed in each of parental, Gα_q_/Gα_11_-null, and arrestin2/3-null HEK293 cells. In certain experiments the Gα_q_ + Gα_11_ inhibitor YM-254890 (*YM*, 100 nm) was added 30 min before the agonist TUG-891. In other studies cells were pretreated with pertussis toxin (*PTX*) overnight before the addition of TUG-891. ERK1/2 phosphorylation was measured at the indicated time points and is presented as -fold over basal. NOTE: the different scale of the *y* axis in *A* and *B*.

In parental HEK293 cells expressing mFFA4-eYFP, the addition of TUG-891 also stimulated ERK1/2 phosphorylation. This signal, which was relatively modest, peaked within 5 min of agonist addition and then decayed to basal levels (Fig. 8*A*). This was mediated by Gα_q_/Gα_11_, as it was lacking when these cells were pretreated with YM-254890 (Fig. 8*A*). The role of Gα_q_/Gα_11_ was confirmed because TUG-891 was also completely unable to promote ERK1/2 phosphorylation in Gα_q_/Gα_11_-null cells expressing mFFA4-eYFP (Fig. 8*A*). By contrast, in arrestin2/3-null cells expressing mFFA4-eYFP TUG-891 also increased ERK1/2 phosphorylation. In these cells signal also peaked at 5 min post-agonist addition and although slightly more sustained than in the parental HEK293 cell background (Fig. 8*A*) was quantitatively no larger at peak (Fig. 8*A*). Interestingly, expression of mFFA4-PD-eYFP in the parental HEK293 cell background resulted in a much more quantitatively robust stimulation of ERK1/2 phosphorylation in response to TUG-891 than produced by the wild type receptor (Fig. 8*B*) (note differences in the scale of the *y* axis in Fig. 8, *A* and *B*). Although also peaking 5 min after the addition of agonist, ERK1/2 phosphorylation remained elevated over at least a 30-min period in this setting (Fig. 8*B*). Once again, however, this was all but attenuated by pretreatment with YM-254890 (Fig. 8*B*). This latter feature suggested that ERK1/2 phosphorylation, which in other systems can be promoted by a range of G protein-dependent and non-canonical, G protein-independent, pathways, was mediated exclusively via activation of G_q_/G_11_ in this cell background. However, although exceptionally modest compared with the effect in wild type HEK293 cells, in Gα_q_/Gα_11_-null cells mFFA4-PD-eYFP was able to cause a <2-fold elevation of ERK1/2 phosphorylation (Fig. 8*B*). Interestingly, this effect was not ablated by treatment with Pertussis toxin (Fig. 8*B*), although in some settings activation of FFA4 can generate signals and functions via pertussis toxin-sensitive, G_i_ family G proteins (16, 32). Finally, we assessed if mFFA4-PD-eYFP would be able to regulate ERK1/2 phosphorylation in the absence of arrestins. Indeed, TUG-891 produced extremely robust activation in this setting (Fig. 8*B*). Therefore, rather than promoting ERK1/2 phosphorylation in the context of HEK293 cells via expressed FFA4, arrestins appear to provide a negative constraint on FFA4 regulation of this end point.

## Discussion

Here we have employed novel CRISPR/Cas9 genome-edited HEK293 cells in which expression of either Gα_q_ and Gα_11_ or arrestin2 and arrestin3 was eliminated, in combination with a phosphorylation-deficient GPCR variant to dissect the relative contributions of G protein-, arrestin-, and receptor phosphorylation-dependent pathways to signaling of the FFA4 receptor. In so doing we have not only revealed an otherwise unappreciated complexity of FFA4 regulation of ERK1/2 signaling but we have provided important validation and characterization of the genome-edited cell lines. We anticipate that these cell lines will become widely used by the community to study the signaling modalities of other GPCRs, because HEK293 cells have been one of the most important model systems for such work over many years.

Although arrestins were named initially for their capacity to arrest or limit the duration and intensity of G protein-mediated signaling by binding to agonist-occupied GPCRs (3) and in so doing prevented simultaneous interactions with heterotrimeric G proteins, in recent years considerable focus has been given to distinct roles of the arrestins to promote overlapping or alternate signaling cascades (3). Observations of effects in cells and tissues of arrestin knock-out mouse lines (5) have provided clear support for roles of arrestins that extend well beyond their initially defined role in receptor desensitization. Despite such understanding, many studies that explore the mechanistic details of such potential non-G protein-mediated effects of agonist actions at GPCRs have centered on the use of model cell systems, not least HEK293 cells (11–14). In substantial part this reflects on the ease of transfection of plasmids designed to either reduce the levels of expression of arrestin isoforms via induced degradation of the corresponding mRNA or to produce “dominant negative” proteins designed to sequester arrestin-interacting proteins. However, by their nature, such approaches can only result in partial and incomplete effects, and this can cloud the interpretation of data. In the current studies we have, therefore, taken advantage of recent revolutions in genome-editing to generate HEK293 cells that lack expression of the ubiquitously expressed arrestin isoforms, arrestin2 and arrestin3. Although it is often assumed that these two isoforms are functionally redundant, it is important to note that evidence is emerging that they may play non-overlapping roles (33, 34). Subsequently, we introduced either mouse or human FFA4 receptor into the arrestin2/3-null cells as well as into parental HEK293 cells and, after isolation of individual clones, then compared responsiveness after the addition of the FFA4 synthetic agonist TUG-891 (25, 26). FFA4 is a GPCR that responds to medium and longer chain free fatty acids and is considered a potential therapeutic target for various metabolic disorders, including type II diabetes (16, 35). It is generally considered as a receptor that couples with high selectivity to the members of the G_q_/G_11_ G protein subfamily to elevate intracellular Ca^2+^ subsequent to phosphoinositidase C-mediated production of inositol 1,4,5 trisphosphate, and as such, we also expressed forms of FFA4 into HEK293 cells that had been genome-edited to eliminate expression of both Gα_q_ and Gα_11_. This was designed to provide controls for G protein-mediated signaling, but as YM-254890 is a very effective and selective inhibitor of these G proteins (28–31), we also employed this reagent to help dissect contributions from these regulators. Importantly, elimination of expression of either Gα_q_ and Gα_11_ or arrestin2 and arrestin3 did not result in significant changes in expression levels of other G protein α subunits or in levels of Gα_q_/Gα_11_ in the arrestin2/3-null cells. It is well appreciated that in mouse gene knock-out models there is potential for some level of compensatory changes in protein expression patterns linked to the sustained lack of specific gene products. This, however, is more likely to be the case in such knock-out animals, in which there are physiologically relevant pressures for adaptation and compensation than in transformed cell lines such as HEK293 cells.

As well as interacting effectively with Gα_q_ and/or Gα_11_, FFA4 also interacts strongly with arrestins in an agonist-dependent manner (21, 22, 26, 27). In large part this relies on the phosphorylation of two clusters of serine and threonine residues located in the intracellular C-terminal tail of the receptor (21, 22, 36). We previously mapped the sites of phosphorylation induced by the addition of the synthetic FFA4 agonist TUG-891 and altered all of these to alanines (21, 22). By so doing, although not entirely eliminated, agonist-induced interaction between FFA4 and arrestins is greatly reduced (21, 22). As such we also expressed a PD variant of mFFA4 in each of parental, arrestin2/3-null and Gα_q_/Gα_11_-null HEK293, cells to explore aspects of similarity or difference between an FFA4 receptor variant that cannot be phosphorylated and the wild type receptor that cannot interact with arrestins simply due to the lack of expression of these proteins.

Importantly for validation of the broader usefulness of these cells, a number of the results obtained were as predicted from previous studies. In the absence of Gα_q_/Gα_11_, the addition of TUG-891 did not elevate the levels of inositol phosphates and, as such, also failed to elevate intracellular Ca^2+^ levels. However, despite this lack of G protein engagement, agonist occupancy of FFA4 did result in conformational changes in the receptor that allowed it to become phosphorylated, which would normally act as a prelude to interactions with arrestins (37). This was measured by immunoblotting samples with antisera that identify specifically FFA4 (either human or mouse) when amino acids Thr^347^ and Ser^350^ become phosphorylated (21, 22). That this occurred in Gα_q_/Gα_11_-null HEK293 cells as well as in parental cells indicates this does not reflect activation of protein kinase C (PKC), although PKC, as well as G protein-coupled receptor kinase 6 (GRK6) has previously been shown to have capacity to cause phosphorylation of FFA4 (36), and both are expressed by HEK293 cells (38). Moreover, in accord with expectations from earlier studies with other receptors (39, 40), a lack of Gα_q_/Gα_11_ did not interfere with agonist-mediated internalization of FFA4 in response to the addition of agonist nor indeed did inhibition of function of these G proteins with the chemical inhibitor YM-254890. The absence of arrestins, however, largely blocked agonist-induced internalization of wild type FFA4, and the same was true for the PD-FFA4 variant, whether expressed in parental or in arrestin2/3-null HEK293 cells.

Questions of the contribution of arrestins to GPCR-mediated phosphorylation and activation of the ERK1/2 MAP kinases have been studied for many years, initiated in significant part by data from HEK293 cells transiently transfected to express the β_2_-adrenoreceptor. In part, this reflects the central role of these kinases as a regulatory hub for cellular signaling from both GPCRs and tyrosine kinase receptors. However, a central element of focus on the ERK1/2 MAP kinases also, in part, reflects the availability of high quality antisera able to detect phosphorylated and, therefore, activated forms of these kinases. This has resulted in many semiquantitative studies in which immunoblots to detect levels of phosphorylated ERK1/2 are compared before and after a cellular challenge. These studies implicated temporally separable early and later waves of ERK1/2 MAP kinase phosphorylation as reflecting G protein and arrestin-mediated engagement, respectively (41). Herein, stimulation of parental HEK293 cells expressing FFA4 resulted in a rapidly produced peak of ERK1/2 phosphorylation, which was modest in extent and returned rapidly to near basal levels. This was clearly mediated largely by Gα_q_/Gα_11_, as it was blocked by preaddition of YM-254890 and was completely lacking in Gα_q_/Gα_11_-null cells expressing the wild type receptor. Arrestin2/3-null HEK293 cells expressing wild type receptor displayed a similarly modest peak and profile of TUG-891-induced ERK1/2 phosphorylation as observed in the wild type HEK293 cell background. Much more notably, in parental cells expressing mFFA4-PD, the addition of TUG-891 resulted in an equivalently rapid but much larger peak of ERK1/2 phosphorylation than in the cells expressing wild type FFA4, which although reduced within 10 min, reached a new plateau level akin to the pattern often attributed to arrestin-mediated signaling. However, this was also all but eliminated by treatment with YM-254890, suggesting it to also reflect Gα_q_/Gα_11_-mediated signaling. Moreover, the much higher immediate peak and sustained plateau of ERK1/2 phosphorylation was also observed in arrestin2/3-null HEK293 cells expressing FFA4-PD. To assess if there was a component of the ERK1/2 activation that was not related directly to Gα_q_/Gα_11_, we closely examined the effects in Gα_q_/Gα_11_-null HEK293 cells. Here, although no response could be detected for wild type FFA4, a modest but detectable effect was recorded in such cells expressing FFA4-PD. To assess the mechanism of this effect, we treated these cells with the G_i_ G protein-family inhibitor pertussis toxin because at least in certain cell types physiologically relevant signals from FFA4 have been shown to be blocked by pertussis toxin treatment (16, 32). Treatment with pertussis toxin was, however, unable to completely eliminate the phosphorylation of ERK1/2 in this setting. This would suggest a Gα_i_-independent mechanism that remains to be elucidated. Previous studies using siRNA-induced knockdown of arrestins in HEK293 cells indicated this to result in enhanced stimulation of ERK1/2 phosphorylation by the endogenously expressed muscarinic M_3_ receptor (38). This implies that different class A GPCRs may well employ different strategies to engage and regulate this key hub of signal integration.

Overall, the results provided herein suggest that in a HEK293 cell background, arrestin engagement with FFA4 does not result in a non-canonical pathway to ERK1/2 activation. Instead, the enhanced signals of FFA4-PD suggest that a lack of phosphorylation and poor engagement with arrestins and, potentially, the very restricted ability of this receptor to internalize results in more extensive and more prolonged G protein-mediated signals that maintain ERK1/2 phosphorylation. To assess this more fully we considered the extent and kinetics of Ca^2+^ signaling as a monitor of potential receptor desensitization. We have previously shown that in HEK293 cells FFA4 desensitizes rapidly to a single pulse of agonist but that this can also be reversed rapidly with agonist washout (26). Herein, we first confirmed rapid and profound desensitization of Ca^2+^ elevation in response to TUG-891 in parental HEK293 cells expressing the wild type mouse receptor. In contrast, in arrestin2/3-null cells, such desensitization did not occur, and repeated pulses of TUG-891 caused subsequent pulses of Ca^2+^ elevation. This was noticeable in the arrestin2/3-null cells, even with a single pulse of TUG-891; whereas the time to peak of Ca^2+^ release was not distinct from that observed in parental HEK293 cells, the time for recovery to basal was markedly extended. Clearly, measured levels of [Ca^2+^]*_i_* reflect a balance between release and re-uptake from intracellular stores. Rapid, indeed almost immediate, FFA4 phosphorylation and desensitization would allow rapid return to basal in the presence of effective re-uptake. By contrast, although re-uptake may be unchanged, limited desensitization in the arrestin2/3-null cells implies ongoing release that will result in elevated levels being maintained for longer. This was markedly extended in both parental and arrestin2/3-null cells. Now, indeed [Ca^2+^]*_i_* levels were maintained at greatly elevated levels for >10 min. This suggests that FFA4-PD, able to interact at best weakly with arrestins and maintained at the cell surface, can continue to signal via Gα_q_/Gα_11_ over a sustained period, and thus the cells are unable to return to a basal equilibrium with maintained low [Ca^2+^]*_i_*_._ That this was not nearly as pronounced in arrestin2/3-null cells expressing wild type FFA4 does suggest, however, that arrestins are not the only contributors to the function and regulation of FFA4 despite it being well established that this receptor interacts in a sustained fashion with arrestins and, in cells with a wild type background, is able to internalize into endosomes in concert with an arrestin (26).

The rapid progress of approaches in genome editing is now primed to offer ways to understand roles of specific components of signaling pathways as well as ways to assess potential off-target effects of activators and inhibitors of proteins within such pathways. The current studies show that, at least in the background of HEK293 cells, arrestins play a more important role in the functions of FFA4 as traditional arresting molecules of canonical G protein-mediated signals than as non-canonical signal initiators, as we have been unable to detect a role for arrestins in promoting ERK1/2 activation after stimulation of the FFA4 receptor.

## Experimental Procedures

### 

#### 

##### Materials

Unless otherwise stated, all chemicals and reagents were from Sigma. Tissue culture reagents and buffers were from Life Technologies Inc. TUG-891 (3-(4-((4-fluoro-4′-methyl-[1,1′-biphenyl]-2-yl)methoxy)phenyl)-propanoic acid) was as described previously (25–27). Cellular signaling assay kits (IP-One and pERK1/2) were obtained from Cisbio Bioassays (Codolet, France). Antisera for the detection of arrestin2 (product number #12697) and arrestin3 (product number #3857) were purchased from Cell Signaling (Nottingham, UK), and anti-α-tubulin antibody (product number T9026) was purchased from Sigma, whereas Gα_q_/Gα_11_ Gα_s_, Gα_i1/2_, and GFP antisera were produced in house. Phospho-site- specific antisera were raised against the peptide GAIFT^(P)^DTS^(P)^VRRND. which corresponds to amino acids 343–357 of mouse (m)FFA4 (22) and KGAILT^(P)^DTS^(P)^VKR for human (h)FFA4 (21). IREDye® 800CW or IREDye® 680 RD secondary antibodies were from LI-COR (Cambridge, UK). Protease and phosphatase inhibitor cocktails were from Roche Applied Science. YM-254890 was a kind gift of Astellas Pharma Inc. (Osaka, Japan).

##### Plasmids and Mutagenesis

All of the FFA4 constructs tagged with eYFP were generated as previously described (21, 22, 26, 27). Briefly, the wild type or PD FFA4 receptors were fused at their C terminus to eYFP and to an N-terminal FLAG epitope and subsequently subcloned into pcDNA3.1 for constitutive expression. The identity of all new constructs generated was verified by nucleotide sequencing. In certain cases eYFP was replaced with monomeric Venus fluorescent protein (mVenus).

##### Generation of Gα_q_/Gα_11_ and Arrestin2/3-null Cells

Generation of HEK293 cells devoid of functional Gα_q_ and Gα_11_ was previously described (23). Expression from the genes for arrestin2 and arrestin3 was also eliminated using a similar strategy that will be reported in detail elsewhere.

##### Cell Lines

Parental HEK293, Gα_q_/Gα_11_-null, and arrestin2/3-null HEK293 cells were maintained in Dulbecco's modified Eagle's medium (DMEM) supplemented with 10% fetal bovine serum (FBS), penicillin (100 units·ml^−1^), and streptomycin (100 mg·ml^−1^) at 37 °C and 5% CO_2_ in a humidified atmosphere. Parental, Gα_q_/Gα_11_-null HEK293, and arrestin2/3-null HEK293 cells were transfected with either 5 μg of the wild type FFA4 receptors (FFA4-eYFP) or the phospho-deficient forms of FFA4 (FFA4-PD-eYFP) plasmids in a 1:6 ratio using polyethyleneimine. After 24 h cells were serially diluted into 10-cm dishes where foci were allow to grow under selection with DMEM supplemented with Geneticin (Promega). Individual clones were isolated and screened for homogeneous eYFP/mVenus expression. A single clone per cell line was selected for subsequent experiments.

##### HTRF-based IP1 and ERK1/2 Phosphorylation Assays

IP1 assays (Cisbio Bioassays) were performed according to the manufacturer's instructions. In brief, a suspension of 7500 cells/well was incubated with the stated concentrations of agonist for 1 h in the presence of 10 mm lithium chloride. IP1 accumulation was subsequently measured using a PHERAstar FS plate reader (BMG Labtech, Offenburg, Germany). Quantification of phosphorylated ERK1/2 levels was performed using the phospho-ERK (Thr^202^/Tyr^204^) cellular assay kit (Cisbio Bioassays). Cells were plated at 45,000 cells/well in poly-d-lysine-coated 96-well plates and on the day of the experiment were incubated with FBS-free medium for 4 h, after which they were treated with compounds and inhibitors for the stated times. After removal of these, 50 μl of lysis buffer was added to each well, and plates were incubated for 30 min on an orbital shaker at room temperature. 16 μl of cell lysates were transferred to a white 384-well proxi-plate and incubated in the dark for 2 h at room temperature with anti-phospho-ERK1/2-d2 (2 μl) and anti-phospho-ERK1/2-Eu^3+^-cryptate (2 μl). Time-resolved fluorescence resonance energy transfer signals were measured using a PHERAstar FS plate reader.

##### Western Blotting

Cell lysates, membrane preparations, or cytosolic fractions were size-fractionated on 4–12% SDS-PAGE gels and transferred to nitrocellulose membranes. Nitrocellulose membranes containing resolved receptor proteins were incubated in phosphate-buffered saline or Tris-buffered saline LI-COR blocking buffer and incubated subsequently with the stated antibody/antiserum dilutions. Membranes were air-dried and scanned using an LI-COR Odyssey CLx Imager (Cambridge, UK) for the detection of polypeptides.

##### Single Cell Calcium Imaging

Cells stably expressing the FFA4-eYFP constructs were cultured on 0 thickness poly-d-lysine-coated glass coverslips for 24 h in complete DMEM. Single-cell Ca^2+^ measurements in cells loaded with Fura-2 AM dye were then performed as described previously (42).

##### Visualization of FFA4 Internalization

Cells expressing the various mFFA4-eYFP/mVenus constructs were cultured on poly-d-lysine-coated glass coverslips and cultured for 24 h. Live cells were transferred to Hanks' balanced salt solution and then imaged using a Zeiss VivaTome spinning disk confocal microscopy system (Karl Zeiss, Oberkochen, Germany). Images on at least 7 z-sections were taken before the addition of ligand and every 5 min after ligand addition for a total of 30 min.

##### Cell Surface ELISA

HEK293 cell variants expressing the different constructs were cultured on poly-d-lysine-coated flat-bottom 96-well plates for 24 h and cultured in FBS-free medium for 4 h and with ligand for the indicated times after which cells were fixed using 4% paraformaldehyde, 0.3 m sucrose solution. These were subsequently incubated in phosphate-buffered saline containing 5% bovine serum albumin to block nonspecific binding sites (30 min at room temperature) followed by incubation with an anti-FLAG monoclonal primary antibody (product number #T6199) (30 min at room temperature) and finally with an anti-mouse horseradish peroxidase-conjugated secondary antibody (30 min at room temperature). Cells were washed 3 times with phosphate-buffered saline before measuring Hoechst 33342 fluorescence using a PolarStar Omega plate reader. After washing a final time with phosphate-buffered saline and incubating with 3,3′,5,5′-tetramethylbenzidine horseradish peroxidase substrate in the dark at room temperature, the absorbance at 620 nm was measured on a PHERAstar FS plate reader. Receptor surface expression was calculated as the 620-nm absorbance corrected for cell number based on Hoechst fluorescence.

##### Data Analysis

All data present represent the means ± S.E. of at least three independent experiments. Data analysis and curve fitting was carried out using the Graphpad Prism software package v5.0b (GraphPad Software, La Jolla, CA). Analysis of images was carried out using either the Image Studio lite v5.2 (LI-COR, Cambridge, UK) or ImageJ/Fiji software (43).

## Author Contributions

G. M. and A. B. T. conceived this work. A. I. generated reagents. E. A.-C., L. J., S. Z. R., and R. P. performed the experiments. G. M., E. A.-C., and A. B. T. wrote the paper with assistance from all authors.

## Footnotes

3 The abbreviations used are:

GPCR: G protein-coupled receptor
eYFP: enhanced yellow fluorescent protein
FFA4: free fatty acid receptor 4
IP1: inositol monophosphate(s)
PD: phosphorylation-deficient
mFFA4: mouse FFA4
hFFA4: human FFA4.
